# An investigation of media reports of digital surveillance within the first year of the COVID-19 pandemic

**DOI:** 10.3389/fdgth.2023.1215685

**Published:** 2023-07-24

**Authors:** Leigha Comer, Lorie Donelle, Marionette Ngole, Jacob J. Shelley, Anita Kothari, Maxwell Smith, James M. Shelley, Saverio Stranges, Brad Hiebert, Jason Gilliland, Jacquelyn Burkell, Tommy Cooke, Jodi Hall, Jed Long

**Affiliations:** ^1^ Arthur Labatt Family School of Nursing, Western University, London, ON, Canada; ^2^ College of Nursing, University of South Carolina, Columbia, SC, United States; ^3^ Western Law, Western University, London, ON, Canada; ^4^ School of Health Studies, Western University, London, ON, Canada; ^5^ Faculty of Health Sciences, Western University, London, ON, Canada; ^6^ Department of Epidemiology and Biostatistics, Schulich School of Medicine and Dentistry, Western University, London, ON, Canada; ^7^ Departments of Family Medicine and Medicine, Schulich School of Medicine and Dentistry, Western University, London, ON, Canada; ^8^ The Africa Institute, Western University, London, ON, Canada; ^9^ Department of Precision Health, Luxembourg Institute of Health, Strassen, Luxembourg; ^10^ Department of Geography and Environment, Western University, London, ON, Canada; ^11^ Faculty of Information and Media Studies, Western University, London, ON, Canada; ^12^ Surveillance Studies Centre, Queen’s University, Kingston, ON, Canada; ^13^ School of Nursing, Fanshawe College, London, ON, Canada

**Keywords:** COVID-19, pandemic, digital surveillance, digital health, public health, technology, mass media

## Abstract

**Introduction:**

The COVID-19 pandemic prompted a surge in digital public health surveillance worldwide, with limited opportunities to consider the effectiveness or impact of digital surveillance. The news media shape public understanding of topics of importance, contributing to our perception of priority issues. This study investigated news media reports published during the first year of the pandemic to understand how the use and consequences of digital surveillance technologies were reported on.

**Methods:**

A media content analysis of 34 high- to low-income countries was completed. The terms “COVID-19,” “surveillance,” “technologies,” and “public health” were used to retrieve and inductively code media reports.

**Results:**

Of the 1,001 reports, most were web-based or newspaper sources on the development and deployment of technologies directed at contact tracing, enforcing quarantine, predicting disease spread, and allocating resources. Technology types included mobile apps, wearable devices, “smart” thermometers, GPS/Bluetooth, facial recognition, and security cameras. Repurposed data from social media, travel cards/passports, and consumer purchases also provided surveillance insight. Media reports focused on factors impacting surveillance success (public participation and data validity) and the emerging consequences of digital surveillance on human rights, function creep, data security, and trust.

**Discussion:**

Diverse digital technologies were developed and used for public health surveillance during the first year of the COVID-19 pandemic. The use of these technologies and witnessed or anticipated consequences were reported by a variety of media sources worldwide. The news media are an important public health information resource, as media outlets contribute to directing public understanding and shaping priority public health surveillance issues. Our findings raise important questions around how journalists decide which aspects of public health crises to report on and how these issues are discussed.

## 1. Introduction

Public health surveillance is defined by the World Health Organization as continued watchfulness and the monitoring of health-related events in humans linked to action (1). While surveillance has a long history in public health as a tool for supporting disease detection, treatment, and prevention, the COVID-19 pandemic has been described as the first pandemic of the “algorithmic age,” where data science analytics increasingly contribute to disease surveillance (2). The pandemic has prompted a surge in the development and deployment of digital technologies used for public health surveillance worldwide. However, urgency to control the spread of COVID-19 and to support other public health goals has limited opportunities to meaningfully consider the effectiveness of digital surveillance in achieving these goals or the intended and unintended consequences related to digital surveillance (e.g., impacts on human rights and civil liberties). Public health and other interest groups have expressed concerns regarding the short- and long-term potential of digital surveillance for undermining human rights, including freedom of expression and freedom of association (3–5).

This study seeks to fill gaps in our knowledge of digital technologies used for public health surveillance during the COVID-19 pandemic through a review of mass media reports published during the first year of the pandemic (from January 2020 to December 2020). The news media serve as a powerful tool for communication, providing information to support people's decision making on a variety of public (e.g., community, social, and political) affairs (6). The news media also shape public understanding of topics of importance, contributing to our understanding of priority issues of the day (7). Salience-based theories hold that the extent of press attention is critical in determining the degree of importance accorded to topics being covered, supporting the idea that readers and viewers of news media perceive issues emphasized by the news media as important (8). Likewise, the news media also play an agenda-setting role in the policy process as they influence which issues, people, and topics are perceived as most important (9).

In the early days of the COVID-19 pandemic, the news media served a particularly important role in conveying information about the disease and recommended protective public health strategies (10, 11). These spheres of influence extended to digital surveillance and the technologies being deployed worldwide for public health surveillance during the COVID-19 pandemic. As such, this study sought to answer the following four research questions: (1) in the first year of the COVID-19 pandemic, which countries were reported by the mass media as using digital technologies for public health surveillance to mitigate the COVID-19 pandemic? (2) according to the news media, what was the nature (intensity, scope) of digital technology use? (3) according to media reports, how did digital surveillance align with public health goals (e.g., identification of cases, disease mitigation)? and (4) according to media reports, what were the intended, unintended, witnessed, and anticipated impacts of digital surveillance on individuals and populations globally? These questions were explored through a content analysis of media reports published during the first year of the COVID-19 pandemic. The findings of this analysis have implications for how news media report on issues around public health and surveillance, how these issues are reported across the world, and the topics prioritized as particularly salient by the news media in the first year of a global public health crisis.

## 2. Methods

During the early months of the COVID-19 pandemic, our interdisciplinary team of researchers selected 34 countries to include in our search and analysis based, in part, on a rapid review of pertinent academic and grey literature (Table 1). Countries were chosen with the aim of ensuring representation across different regions and across high-, middle-, and low-income countries based on rankings by the World Bank (12). The interdisciplinary perspectives of members of our research team also supported our objective of preparing a sample of countries that represent a variety of geopolitical contexts, different responses to the COVID-19 pandemic, varying impacts of COVID-19 (e.g., case number, mortality rates), and novel or interesting uses of digital technologies for public health surveillance. In larger regions, e.g., Asia, we strove to achieve representation of different subregions, e.g., Eastern Asia, Southeast Asia, etc. Our categorization of countries into different regions took into account World Bank and World Health Organization groupings as well as regional classifications used by the Nexis Uni and Factiva databases (13).

**Table 1:** 34 countries included in our sample.

Africa	Asia	Oceania	Caribbean Islands	Central and South America	Europe	North America
Ghana	China	Australia	Dominican Republic	Brazil	France	Canada
Rwanda	India	New Zealand	Haiti	Chile	Italy	Mexico
South Africa	Iran	Pacific Islands	St Lucia	Panama	Romania	United States of America
Tunisia	Israel				Spain	
Uganda	Russia				Sweden	
	Saudi Arabia				United Kingdom	
	Singapore					
	South Korea					
	Taiwan					
	Vietnam					
	Yemen					

### 2.1. Data collection

Working with a research librarian, we developed a search strategy to capture all media reports relevant to our research questions while limiting the number of irrelevant search results. The search syntax was trialed by searching two databases recommended by the research librarian: Nexis Uni and Factiva. Results of these initial searches were shared with the research team to further refine our search terms. Through this process, a final search strategy was designed with the following search terms: ((“population surveillance” OR “public health surveillance” OR surveillance OR “digital surveillance” OR “biosurveillance” OR “surveillance technology” OR “surveillance technologies” OR “epidemiological monitoring”) AND (“pandemic” OR “disease outbreak” OR “coronavirus infections” OR “covid19” OR “covid-19”)) AND (“public health” OR “public health applications”).

Using these search terms and syntax, two researchers conducted a search of Nexis Uni and Factiva in March and April 2021 to collect mass media reports. The search was limited to reports published or broadcast between January 2020 and December 2020 to capture the first year of reporting on the COVID-19 pandemic. Media reports from all sources were considered for inclusion if they could be retrieved in text form, e.g., newspaper articles, magazines, blog posts, and transcripts from radio and television broadcasts. We included reports in languages other than English by using the databases' automated internal translation tools to translate reports that were not published in English. The search was further limited by including only media reports that mentioned digital surveillance during the COVID-19 pandemic conducted within the 34 countries included in our sample.

This search strategy yielded 36,526 results from Nexis Uni and 37,450 results from Factiva. Two researchers screened these results and retained documents for analysis if they met all the following inclusion criteria:

• Mentions use of a digital technology for public health surveillance by one of the 34 countries included in our sample.
• Public health surveillance addresses the COVID-19 pandemic.
• Public health surveillance focuses on monitoring humans rather than non-human animals.

Following screening, 971 results were retained from Nexis Uni and 849 results were retained from Factiva. After combining these results and removing exact duplicates (i.e., identical titles and text content published or broadcast by identical sources), we retained a total of 1,001 documents for analysis. At all points during this screening process, in cases where the researchers disagreed or were uncertain whether a document met the inclusion criteria, a third researcher read the full text and, following discussion among the researchers, determined whether to retain the text for analysis.

**Figure 1:**
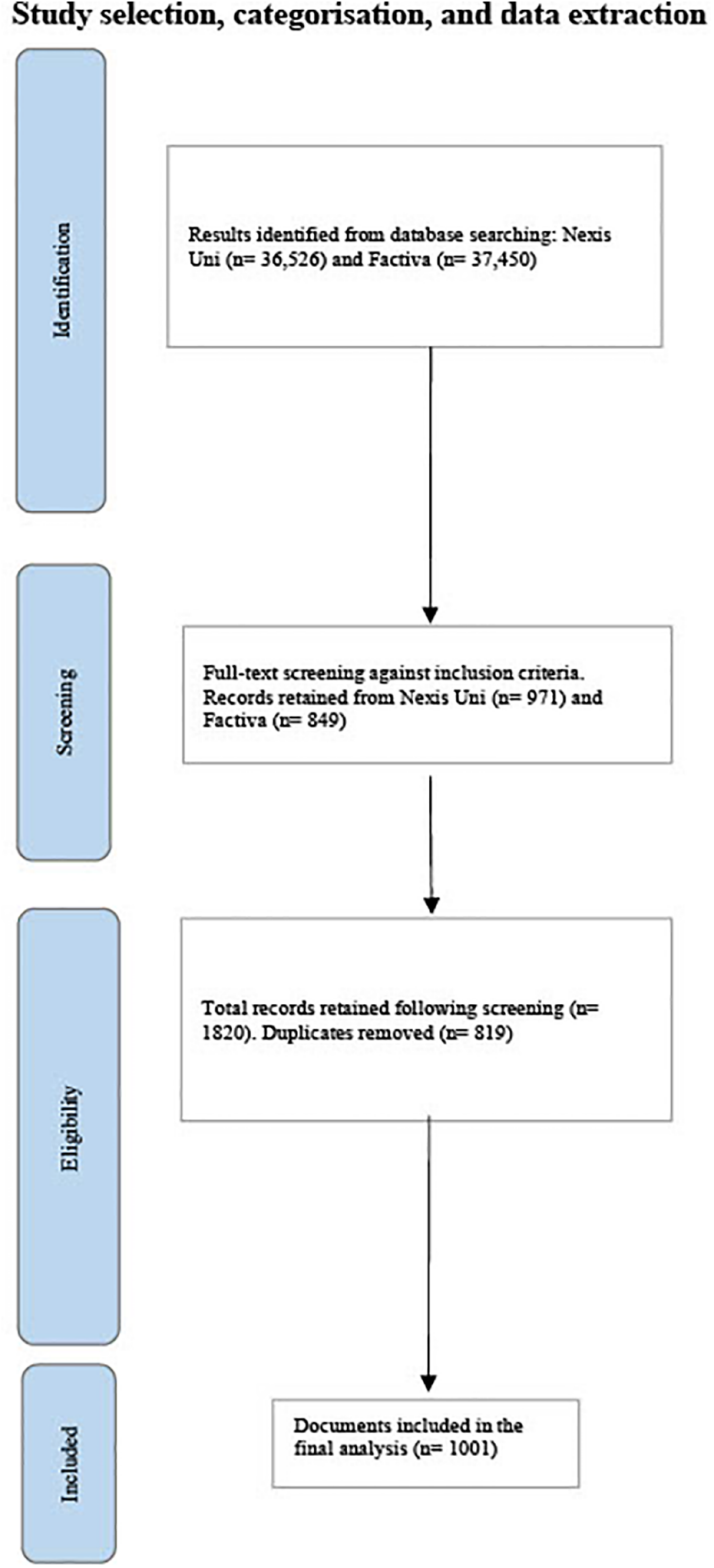
PRISMA diagram detailing the study selection process.

See Figure 1 for the PRISMA chart detailing the study's inclusion process.

### 2.2. Data analysis

Data extraction occurred in two phases. First, given the large number of retained documents, a data extraction table was created to obtain an overview of digital technologies used for public health surveillance during the COVID-19 pandemic across the 34 countries in our sample. Two researchers developed, trialed, refined, and used the table to extract the following information from each media report: countries identified in the report; digital technologies used for public health surveillance and their application(s); date of publication; and word count. For each media report analyzed, we coded information regarding the source, including source format (e.g., newspaper, radio broadcast), title of the source of publication or broadcast (e.g., the National Post, National Public Radio), date of publication, and location of publication (i.e., country in which the text was published or the broadcast occurred). This initial data extraction phase informed the creation of a coding framework for content analysis, including codes related to: media source; country in which digital technology was used for surveillance; type of digital technology used for public health surveillance; witnessed and predicted consequences of digital surveillance; and whether digital surveillance was reported as successful or unsuccessful for achieving public health aims. Coding was facilitated using NVivo software. Two researchers piloted an initial set of codes and then discussed identified themes with three other researchers. Content analysis through inductive coding was conducted on each document by two researchers (14, 15).

While we primarily focused on digital surveillance in the countries included in our sample, we also coded instances of digital surveillance in all countries mentioned in the retained reports to gain an overview of the full breadth of digital surveillance worldwide. Other countries in which at least one instance of digital surveillance during the COVID-19 pandemic was mentioned included Kuwait, Estonia, Indonesia, Qatar, Ireland, Belgium, Thailand, Nigeria, Hong Kong, the Netherlands, Malaysia, Switzerland, Germany, and Japan.

Findings were shared and discussed with our interdisciplinary team of researchers for analysis. Iterative data collection and analysis ensured that interdisciplinary insight was applied to the findings from a range of expert perspectives, including nursing, medicine, public health, epidemiology, surveillance studies, geography, health information science, law, bioethics, policy, knowledge translation science, critical theory, and sociology. This systemic approach of engaging multiple coders and researchers was adopted to attend carefully to instances of disagreement among researchers throughout data analysis as a means of flagging potential biases and differing perspectives. In particular, discussions of diverging perceptions were key to thematic analysis as we drew on the multidisciplinary perspectives of the research team. Many times, disagreements among researchers regarding data analysis were critical to guiding fruitful discussions that allowed for uncovering the diverse ways researchers' different disciplinary approaches led to diverging understandings of the findings and emphases on which findings were most important or meaningful. Furthermore, we ensured data analysis was rigorous through techniques suited to qualitative research, including ensuring dependability by comparing researchers' analyses and identifying potential biases through weekly reflexive discussions (16). Regular discussion of the findings and analyses also ensured consistency in data analysis and shared understanding among the researchers.

## 3. Results

Most of the media reports retained for analysis were web publications (e.g., online blogs and news sites) (*n* = 415), followed by publications from newspapers (*n* = 384), newswire services (*n* = 179), magazines (*n* = 45), newsletters (*n* = 21), reports from think tanks, academic institutions, and non-profit organizations (*n* = 15), transcripts of television broadcasts (*n* = 12), and transcripts of radio broadcasts (*n* = 9). Reports ranged in length from 204 words to 24,118 words. All reports were written or broadcast in English except two Spanish-language reports published in Spain, which were translated using the internal automated tools described above.

The media reports in our sample came from a total of 365 distinct news sources. We retained media reports published or broadcast in most of the 34 countries included in our sample (except Brazil, Chile, the Dominican Republic, Haiti, Italy, the Pacific Islands, Panama, Romania, Sweden, and Tunisia). Most reports were published or broadcast in the USA (*n* = 423), the UK (*n* = 182), India (*n* = 95), Canada (*n* = 66), and Australia (*n* = 51).

**Figure 2:**
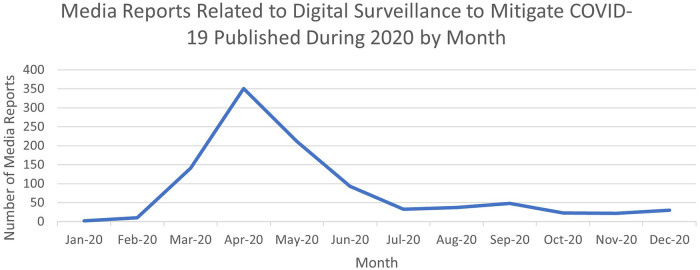
Chart plotting the number of media reports related to digital surveillance to mitigate the COVID-19 pandemic published from January 1, 2020 to December 31, 2020, as yielded through our search.

Most media reports in our sample were published or broadcast in the months of April (*n* = 351), May (*n* = 211), March (*n* = 141), and June (*n* = 95). The months with the fewest reports included January (*n* = 2), February (*n* = 10), November (*n* = 22), and October (*n* = 23). See Figure 2 for a chart plotting the number of reports published or broadcast by month from January to December 2020. As demonstrated in Figure 2, a large majority of the media reports were published or broadcast in the first few months following the declaration of the pandemic in March 2020: 715 reports were published or broadcast by the end of May 2020, representing 71% of our sample. Many reports broadcast or published in March, April, and May 2020 reported on the development and deployment of digital technologies following the declaration by the World Health Organization on March 11th, 2020 that COVID-19 was a global pandemic. As such, most reports described: (1) the development of technologies in response to growing concern around COVID-19 and the pandemic (*n* = 334), and (2) the initial deployment of technologies, including reports on early obstacles and errors in the technologies as they were introduced (*n* = 485).

Our thematic findings provide insight into the types of digital technologies used for public health surveillance, the reported success of these technologies, and the witnessed and anticipated consequences of digital surveillance. The three themes developed through our analysis include: (1) digital technologies used for public health surveillance, (2) success of COVID-19 digital surveillance (with three subthemes: defining success, public participation, and data validity), and (3) the predicted and witnessed consequences of digital surveillance.

### 3.1. Digital technologies used for public health surveillance

The media reported a wide variety of digital technologies used for public health surveillance during the COVID-19 pandemic in the 34 countries sampled. Some of the most frequently identified technologies were mobile applications (e.g., applications to support contact tracing) (*n* = 626), location tracking technologies (e.g., Bluetooth and GPS used for location tracking) (*n* = 269), CCTV cameras and other security cameras (*n* = 103), and facial recognition technology (*n* = 91). There were also reports identifying the use of digital purchase tracking (*n* = 73), aggregated data from telecommunications companies (*n* = 68), digital “smart” thermometers and thermal cameras (*n* = 65), wearable devices (*n* = 62), aggregated and anonymized movement data provided by private companies (*n* = 61), QR codes (*n* = 55), drones (*n* = 48), social media and web search data (*n* = 40), digital passes, travel cards, passports, and certificates (*n* = 31), geofencing technology (*n* = 29), and traffic or transport data (*n* = 15). Among the numerous mobile applications identified, many countries were reported as using the Apple–Google Application Programming Interface (API) to support their mobile contact tracing applications (*n* = 199).

**Figure 3:**
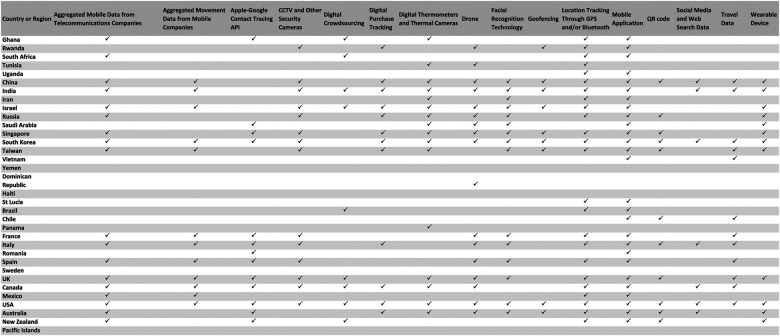
Table indicating which digital technologies were used in which countries and regions, as reported by media reports from the first year of the COVID-19 pandemic included in our analysis.

Of the 34 countries and regions included in our sample in which at least one digital technology was used for public health surveillance during the COVID-19 pandemic, the USA was the most frequently mentioned (*n* = 311), followed by China (*n* = 283), the UK (*n* = 202), South Korea (*n* = 181), and Singapore (*n* = 167). See Figure 3 for a table indicating the types of digital technologies used in each region.

### 3.2. Success of COVID-19 digital surveillance

The media reports identified several applications of digital public health surveillance during the COVID-19 pandemic. The most frequently reported aim of digital surveillance was to support COVID-19 contact tracing (*n* = 476). Other reported uses included enforcing quarantine (*n* = 162), modelling and predicting disease spread (*n* = 79), identifying disease clusters and hot spots (*n* = 64), real-time monitoring of disease spread (*n* = 58), supporting social distancing (*n* = 54), measuring the effectiveness of public health measures (*n* = 47), informing public health interventions (*n* = 47), and supporting the direct provision of health care (*n* = 40).

Some of the technologies considered successful in achieving the abovementioned outcomes included mobile applications (e.g., use of a mobile applications to support contact tracing by Singapore, Switzerland, Australia, China; use of a mobile application to enforce quarantine of infected individuals by South Korea), location tracking through mobile phones (e.g., in South Korea, China, Israel, Singapore, Taiwan), CCTV cameras (e.g., in South Korea, Israel, Taiwan), and tracking of purchases and transactions (e.g., in South Korea, China).

Overall, journalists tended to describe digital surveillance in Asian countries as successful much more frequently than digital surveillance in other countries and regions: while 81 media reports described digital surveillance in at least one Asian country as successful, only 19 reports indicated that digital surveillance was successful in North American countries, and only 11 reports considered digital surveillance successful in European countries. This may be due, in part, to early comparisons between countries; of the 81 reports in which journalists ascribed success to digital surveillance in Asian countries, in 75 reports, they did so as a means of comparing early adoption of digital surveillance in these countries to comparatively later use of digital technologies in other countries as a means of explaining Asian countries' successes at curbing disease spread.

Several Asian countries (i.e., Taiwan, South Korea, Singapore) were also singled out as particularly successful in the use of digital technologies for public health surveillance given their robust public health systems. For instance, some reports (*n* = 12) credited Asian countries' strong public health systems to their experiential learnings from the 2003 SARS outbreak, as journalists contended that the experience of earlier public health crises prompted governments in Taiwan, Singapore, and South Korea to pass laws and to establish public health measures aimed at preventing similar outbreaks in the future. Others (*n* = 6) attributed the success of countries including China and Taiwan to forms of digital surveillance they deemed highly intrusive, authoritarian, and ultimately incompatible with the democratic ideals of “Western” nations, such as the USA.

While relatively few journalists reported the outcomes of digital health surveillance, many drew on expert opinions, existing academic and grey literature, and early examples of surveillance in Asian countries described above to predict whether digital surveillance might be successful and the factors that might impact success. These factors are described below.

#### 3.2.1. Public participation

Of the factors identified as contributing to the success of digital surveillance for public health purposes, many media reports (*n* = 158) addressed the importance of individuals' uptake of digital surveillance strategies (e.g., mobile applications) and raised concerns that a lack of participation across populations might limit their effectiveness. In some reports (*n* = 11), journalists used the term “digital divide” to refer to unequal access to digital technologies (and/or their benefits) due to poverty, limited digital literacy, older age, disability, etc., which may limit participation. Contact tracing applications, among the most widely used surveillance measures globally during the COVID-19 pandemic, were identified (*n* = 38) as limited in value for public health surveillance given their exclusion of people who do not have access to a smartphone or the Internet, including vulnerable groups who have been disproportionately impacted by the COVID-19 pandemic (e.g., people living in poverty and older adults).

#### 3.2.2. Data validity

In many reports (*n* = 154), journalists also questioned the validity of data collected through digital technologies. A frequent concern was the reality that many participatory forms of surveillance rely on self-reporting. For instance, skepticism was expressed toward digital crowdsourcing tools, including online screening questionnaires used in India and the UK, for their use of self-reported COVID-19 symptoms for modelling and predicting disease spread. Errors in digital technologies were also reported. A mobile application used in Russia to enforce quarantine, for instance, was found to have technical flaws that incorrectly flagged users for allegedly breaching self-quarantine, resulting in fines. Similar errors were also reported in an Israeli mobile application to enforce quarantine. Furthermore, there were concerns that reported that digital technologies may conceal or misrepresent social realities, illustrated, for example, by the mobile applications and location tracking technologies used to support contact tracing in West Africa. The accuracy of the data derived from these technologies was questioned, given that West Africans frequently own multiple mobile phones and share phones with family, friends, and neighbours. Public health decisions that do not account for contextual factors may not adequately address the ways people use technologies, how they comply with public health measures, or how disease spreads in these communities.

Numerous reports (*n* = 141) also addressed the limitations of technologies and questioned the value of the data collected. For example, while many location tracking technologies and mobile contact tracing applications—including the Apple–Google API—rely on Bluetooth technology, Bluetooth's limited accuracy may hinder its value for supporting contact tracing efforts. Likewise, while in many countries (e.g., China, India, Russia, South Korea, the UK, and the USA) thermal cameras were used to detect fevers, temperature screening may not be effective in detecting infection, as many people demonstrate diverse COVID-19 symptom expression and not everyone with an elevated temperature is infected with COVID-19. In some reports, journalists (*n* = 13) also remarked that digital surveillance may lead to paranoia or panic. In the case of mobile applications that use Bluetooth technology to identify contacts between people, the inaccuracy of this technology and the potential for false alerts—e.g., registering contact between two people separated by a wall—could create panic or prompt people to be tested when they have not actually been exposed to the virus.

### 3.3. Emerging consequences of digital surveillance

Journalists drew on expert opinions, historical examples of surveillance (e.g., the intensification of surveillance following the 9/11 crisis), comparisons to perceived early adopters of digital surveillance (i.e., Asian countries described above), and witnessed consequences of digital surveillance in identifying numerous emerging consequences of digital surveillance. These included infringements on privacy and other human rights and civil liberties, function creep, thwarting other public health measures, compromised data security, erosion of public trust, and consequences associated with private sector involvement in surveillance.

#### 3.3.1. Perceiving digital surveillance as a trade-off

In several reports (*n* = 177), journalists framed the use of digital technologies as a trade-off between human rights and civil liberties, such as the right to privacy, and mitigating disease spread. Journalists questioned whether the use of intrusive technologies that invaded privacy—e.g., location tracking technologies—could be justified if their use reduced disease spread, thereby ending lockdown measures and allowing people greater flexibility to pursue everyday activities. Journalists also discussed other potential consequences of digital surveillance, including infringing on other human rights and civil liberties and compromising data security, as consequences that might be justified if the “tradeoff” was mitigating the impact of COVID-19 or ending intrusive public health measures.

Furthermore, in numerous reports (*n* = 74), journalists raised concerns that digital surveillance might thwart other public health measures intended to mitigate the COVID-19 pandemic. Some reports (*n* = 19) identified the large number of resources required to support digital surveillance (e.g., financial, digital infrastructure, human resources) and questioned whether these resources might be of greater value if allocated to other public health measures, such as traditional contact tracing, testing, and supporting self-isolating individuals. In some reports (*n* = 25), journalists warned that digital surveillance may create a false sense of security. For example, in the case of mobile applications used in countries with low testing rates—e.g., the USA—if people infected with COVID-19 are not tested, they will not report their infection through a mobile application, and others they have come into contact with will not receive an alert and, therefore, may assume they can safely interact with others.

#### 3.3.2. Infringements on human rights and civil liberties

Among the potential impacts of digital surveillance for human rights and civil liberties, breach of privacy was the most frequently discussed possible adverse effect (*n* = 400). Concerns around privacy were most often raised toward use of digital surveillance technologies in countries including China, South Korea, India, Israel, and Singapore. For instance, in China, location tracking through GPS and Bluetooth, use of mobile applications to track movement and to restrict access to public spaces, surveillance cameras (including cameras mounted outside people's front doors), and digital purchase tracking were described as highly invasive measures that could compromise individuals' right to privacy. In South Korea, privacy concerns largely centered around the integrated use of mobile phone data (including location tracking data through GPS and Bluetooth), credit card purchase tracking, and facial recognition software to monitor people's movements and to support contact tracing. In Israel, the state's repurposing of mobile-phone-based location tracking technology to support contact tracing—usually reserved for anti-terrorism operations—was described by several journalists as highly invasive of individuals' privacy.

Journalists also expressed concerns around the effects of digital surveillance on other human rights and civil liberties, including infringements on individuals' right to freedom of movement. There were numerous reports (*n* = 77) identifying potential and witnessed restrictions on movement associated with digital technologies (e.g., mobile applications, location tracking tools, geofencing technologies) used in countries including Chile, China, Israel, New Zealand, Russia, South Korea, and Taiwan to enforce lockdowns and self-quarantining by forcing individuals to remain at home. In many reports (*n* = 66), journalists also warned that digital surveillance may lead to, or had already prompted, inequitable surveillance or targeting of marginalized groups. For instance, reports on the addition of new security cameras at the USA–Mexico border raised concerns that while these cameras were being installed with the purported aim of decreasing the spread of COVID-19 into the USA, the increase in cameras was connected to augmented measures to limit the entry of migrants from Mexico. Likewise, journalists describing the use of COVID-19 testing data by police in countries such as Canada warned that police access to these data could disproportionately impact Black and Indigenous people. Furthermore, in descriptions of facial recognition technology used in airports and other public spaces to support contact tracing and to verify compliance with public health measures (e.g., masking), some journalists noted that facial recognition algorithms tend to misidentify racialized non-white people.

#### 3.3.3. Function creep

Many reports (*n* = 264) included observations that digital surveillance could lead to function creep: the use of digital technologies or data collected through digital surveillance for reasons other than supporting public health or addressing the COVID-19 pandemic. Just as forms of surveillance introduced by the USA (and other countries) following the September 11, 2001 attacks have continued to persist long after the state of emergency, so too did some journalists propose that digital surveillance leveraged to mitigate the COVID-19 pandemic may not be easily dismantled and that data collected through digital surveillance during the COVID-19 pandemic may be repurposed for other means. For instance, journalists interviewed critics in India who questioned the reluctance of the state to provide a timeline indicating when data collected through the government-sponsored mobile contact tracing application would be permanently deleted. Likewise, journalists questioned why the city of Moscow intended to store tracking data from a mobile application to enforce quarantine for a year, and worried that these data might be used to expand the Russian surveillance regime. Media reports also included interviews with privacy and human rights experts who advised that data collected through digital surveillance might be repurposed to track protesters and their contacts, for profit by private companies, or to expand government surveillance.

#### 3.3.4. Implications for data security

Given the rapid deployment of digital technologies and, in several cases, a paucity of information around how data are collected, shared, and stored, many reports (*n* = 145) included warnings that the security of data collected through digital surveillance could be compromised. In discussing surveillance technologies, journalists posed questions around data security that were as-of-yet unanswered, including: what data will be collected? Will individuals be identifiable? Who will have access to the data? How will data be secured? Who has oversight? Journalists also noted that forms of digital surveillance utilizing centralized databases are particularly vulnerable to security breaches. While countries including Singapore, France, the UK, South Korea, Israel, and Taiwan were reported as using centralized databases to store data gathered through location tracking and mobile applications—due, in large part, to the value of using these aggregated data to identify trends in disease spread and to measure the effectiveness of public health measures, among other uses—some journalists noted that storing data in a centralized database might present too great of a security risk despite the potential value of such a database for public health purposes.

#### 3.3.5. Impacts on trust

Media reports (*n* = 108) drew on comments from the general public as well as expert opinions to suggest that digital public health surveillance may erode public trust. Discussions of trust were highly context-specific: in the case of the USA, for instance, some reports (*n* = 17) noted that trust in government, surveillance, and the technology industry was already declining prior to the COVID-19 pandemic, and that the introduction of new surveillance measures (e.g., mobile applications, security cameras, and location tracking to support contact tracing) had further aggravated existing skepticism. In the case of Australia, some reports (*n* = 8) included warnings that a state-sponsored mobile application used to support contact tracing may struggle with low participation as Australians had little trust in government guarantees that their data privacy would be respected. In the UK, reports (*n* = 8) linked errors in a government-sponsored mobile application to support contact tracing and concerns around data privacy to the ongoing erosion of public trust.

In some cases (*n* = 13), journalists observed that the erosion of trust associated with digital surveillance may deter people from following public health measures, such as masking, testing, and self-isolating. In South Korea, for instance, the travel histories of individuals infected with COVID-19 were tracked through purchase tracking and GPS location tracking through users' mobile phones. These details were then shared through widely disseminated mobile phone alert messages. The detailed nature of the alerts made it possible to identify individuals, leading to the stigmatization of people who were publicly identified. In some cases, publication of location history, including visits to gay bars, led to the identification of queer people who were forcibly outed. Some reports (*n* = 10) drew on comments from public health officials and other experts who worried that fear of homophobia and other reprisals had an unintended consequence of discouraging people from seeking testing or being truthful during interviews with contact tracers.

Media reports (*n* = 81) also highlighted the potential consequences associated with private sector involvement in digital surveillance during the COVID-19 pandemic. These discussions centered around the private sector's existing history of intrusive surveillance and the risk that private companies might attempt to profit from data collected for public health rather than commercial reasons. For instance, in some reports (*n* = 6), journalists criticized partnerships between the NHS (England's publicly funded health care system) and Palantir, a US-based company. To support the NHS in disease modeling and prediction through the use of AI algorithms, Palantir was provided access to patient data (e.g., contact details, race, occupation, gender, physical and mental health conditions, religious and political affiliations, and past criminal offenses). These journalists called for the UK government to reconsider this partnership by noting Palantir's history of surveillance in law enforcement and immigration in the USA and by pointing out that the company might profit in some way from access to large amounts of patient data.

Likewise, in some reports (*n* = 36), journalists raised doubts around the Apple–Google API to support contact tracing given the profit motives of both companies and the risk that they might profit from collecting users' data in some way. The data privacy and security practices of these companies were also called into question: one journalist provided a detailed history of Google's questionable history of data collection, sharing, and use (e.g., illegally collecting children's personal information through YouTube, sharing users' information without obtaining consent, and illegally spying on mobile clients' browser histories) in cautioning against using the Apple–Google API.

## 4. Discussion

This analysis focused on media reports published and broadcast during the first year of the COVID-19 pandemic within a sample of 34 countries to investigate how the news media reported on digital surveillance for public health purposes. Through our search, we identified over a thousand reports from web publications, newspapers, newswire services, and other sources that described the use of technologies including mobile contact tracing applications, location tracking technologies, CCTV cameras, facial recognition technology, purchase tracking, aggregated data from private companies, thermal monitoring, wearable devices, and other digital tools in most of the countries in our sample. In our thematic content analysis, we identified themes related to the success of digital surveillance, factors impacting success, and the potential and witnessed consequences of digital surveillance.

The news media play a powerful role in public communication and education not only by providing information, but also through their agenda-setting function as they shape public perceptions of the salience and importance of various topics (6–9). Through their storytelling function, the media also have the capacity to cultivate certain beliefs, perspectives, values, and attitudes, thereby shaping culture and perceptions of the world (17). In the case of the COVID-19 pandemic, researchers have suggested that the media have shaped public risk perception (18), trust and mistrust (19), fear (20), and compliance with public health measures (11, 21). For the general public, media reporting on COVID-19 has allowed for the swift dissemination of information in ways that are more accessible than research findings, which often take significant time to produce and are written for a select audience. In this way, for many people, the media have been a primary means of accessing information about COVID-19. This is particularly important to consider within the context of the first year of the COVID-19 pandemic, given the paucity of information regarding the disease at that time.

The power of the media to influence which topics of the day are perceived by the general public as important and how issues are discussed must be understood within the context of the ongoing monopolization, privatization, and polarization of the news media (22–24). In the case of the COVID-19 pandemic, as Fuchs (2021) argues, times of crisis are not only times of social change but also times where “communication and communication technologies matter in the production, dissemination and challenge of ideologies” (p. 16) (25). Research on news stories about COVID-19 have highlighted the ways in which news outlets have politicized various aspects of the pandemic, including public health measures, such as vaccination and masking (26, 27). Studies that identify the political slant of news sources have found that consuming reports from partisan news sources shapes individuals' perceptions of the risk of COVID-19 and their willingness to comply with public health measures (28–30). As concerns grow regarding misinformation and the polarization of opinions and beliefs driven, in no small part, by the news media (31–33), there is an urgent need to examine which topics are covered by the media and how various issues are discussed. Furthermore, given the contentious nature of surveillance and potential apprehension among many populations toward surveillance for public health and other purposes, it is crucial to consider how journalists reported on digital surveillance during the pandemic and how this reporting might continue to shape public perceptions of health surveillance (34, 35).

Our analysis focused on a sample of 34 countries chosen with an eye toward ensuring a diversity of geopolitical contexts and experiences with COVID-19. While the media reports we examined contained many mentions of digital surveillance in certain countries (the USA, China, the UK, South Korea, and Singapore, for instance), there were no reports of surveillance in Yemen, Haiti, Sweden, or the Pacific Islands, and only minimal mention of countries in regions including Africa, South America, and the Caribbean Islands. As described below, this may be due to our use of English search terms, which may have limited our ability to retrieve reports from certain from regions. It is also unclear whether this gap is due to a lack of reporting on digital surveillance occurring during the first year of the pandemic in these countries or because digital surveillance did not actually occur in these regions. There has been some research, for instance, on the exclusion of certain countries, populations, and cultures in the media, which occurs on both a local and national level and is perpetuated by a globalized, liberalized, and privatized media (36, 37).

Media representations of digital surveillance worldwide differed not only in regard to which countries were mentioned at all but also how surveillance in different countries was described and depicted. For example, while journalists tended to attribute the success of several Asian countries at curbing disease spread to the use of intrusive, undemocratic modes of surveillance that would not be tolerated in “Western” nations, such as the USA, some researchers have also observed that these representations in Western media draw upon forms of techno-Orientalism that stereotype Asian nations as technologically advanced but morally and intellectually primitive (38, 39). There is a need for further research to ascertain the full scope of surveillance worldwide and to better understand how the media report on digital surveillance and technological innovation in certain regions compared to others.

Also significant are the types of digital technologies used for public health surveillance discussed in the news media. While the media reports we examined overwhelmingly focused on mobile phone applications, and particularly contact tracing applications, there have been questions regarding the value of these technologies given that many applications were discontinued shortly after their deployment or had a minimal impact on curbing disease spread (40–45). In contrast, other, more ubiquitous and widely used forms of public health surveillance—such as syndromic surveillance systems that draw on electronic medical records and health information systems—were not discussed. This may be due to the importance of sensationalism and salience for news media to attract audiences (46), particularly in the face of intense competition among news outlets and the pressures of commercialization (47). Researchers have highlighted the tensions journalists face between maintaining objectivity and neutral information sharing vs. meeting financial goals within the constraints of increasingly privatized news organizations (48, 49). In brief, while other forms of digital surveillance may have had a greater role in collecting, storing, and using individuals' health data throughout the COVID-19 pandemic, these forms of surveillance may not have sufficiently met the criteria of salience—attention, prominence, and valence—to be reported on by the news media. Further research on salience and reporting of issues related to COVID-19 is critical given the important role of the media in shaping what information the public and policymakers consider to be important and, consequently, what is considered to be unimportant or not mentioned at all. This is particularly true in the case of public health crises, such as the COVID-19 pandemic and other emerging infectious disease (e.g., mpox and H5N1), as the news media play a fundamental role in shaping our health and political agendas.

The news media are shaped by numerous sources of bias—including editorial preferences, political leanings, journalists’ perspectives, and financial interests (50–53). Groeling, in his review of empirical research on partisan news, has suggested that the study of media bias requires overcoming challenges including subjectivity and a lack of baselines against which to assess bias (54). Others have proposed that the concept of “media bias” is poorly understood, and that allegations of slant (e.g., charges of bias in “Democratic” vs. “Republican” media) are often not supported by empirical investigations (55, 56). Individuals' perceptions of bias have also been found to depend on a wide range of personal and interpersonal factors, such as political involvement (57). Furthermore, dominant ideological biases circulated by the news media and the factors shaping bias differ significantly between countries and cultures (58–60). Given the complexity of the field and our focus on an international context, it was not feasible within this study to assess the potential bias of the media reports analyzed. However, there is a need for further research in the area of infectious disease reporting in particular to consider the impacts of sources and perceptions of media bias on reporting. This research may benefit from focusing on more local contexts to carefully discern the unique cultural, political, social, and economic factors that influence the news media.

### 4.1. Limitations

As our analysis draws on media publications and broadcasts, we are limited to reporting technologies, forms of digital surveillance, and implications of surveillance as described in these reports. As such, any errors in journalists' reporting will be replicated in our findings.

Given the large number of results yielded by our search strategy, we limited our analysis to a sample of 34 countries. While this sample was carefully selected by our interdisciplinary team of researchers to account for a wide range of geopolitical environments and experiences during the COVID-19 pandemic, it also represents only a portion of global engagement in digital surveillance during the COVID-19 pandemic. This limitation may be mitigated, in some part, by our decision to code all countries identified in the reports we retained for analysis beyond those included in our initial sample of 34 countries.

Our analysis focused on media reporting during the first year of the COVID-19 pandemic as we directed our attention to the agenda-setting role of the media and how digital surveillance was discussed by the news media during this first crucial year of the crisis. However, this focus excludes reports published and broadcast since that time. This limited focus may account, among other things, for the comparatively small number of reports analyzed that described the outcomes of digital surveillance. Furthermore, as we used only two databases (Factiva and Nexis Uni), this may have limited our analysis if certain media sources were not included in either of these databases.

Although we modified our search settings to include publications in all languages, we retained only two reports written in a language other than English. After consulting with a specialist research librarian, we suspect that this may be due to our use of English search terms that did not capture keywords, titles, or abstracts written in languages other than English. This almost exclusive focus on English-language reports may limit our analysis and may account for the lack of findings of digital technology use in certain countries included in our sample (e.g., Sweden). Furthermore, while we relied on automated internal translation tools through Factiva and Nexis Uni, we did not verify the accuracy of the translations.

Finally, while a strength of this study is its global focus on media reporting in 34 different countries, a limitation of this broad focus was our inability to attend in greater detail to the unique political, economic, social, or cultural contexts of these countries and how these factors might shape media reporting and digital public health surveillance. As noted above, future research may benefit from focusing more narrowly on local or regional contexts to analyze dimensions of media reporting and surveillance that are unique to specific countries and regions.

## 5. Conclusion

In this analysis of media reporting during the first year of the COVID-19 pandemic, we explored the use of digital surveillance technologies for public health purposes in 34 countries around the globe. Through our descriptive analysis, we identified the regions, types of technologies, and potential and witnessed consequences associated with digital surveillance that were most frequently reported in the news media. These findings were considered in the context of the role of the news media in agenda-setting, information sharing, and cultivating certain beliefs, values, and perspectives. These findings raise important questions around how journalists decide which aspects of public health crises to report on, how these issues are discussed, and the impacts of reporting on shaping individuals' perspectives of the COVID-19 pandemic and digital surveillance for achieving public health aims. Although this analysis focused on the first year of reporting during the COVID-19 pandemic, the findings have implications for reporting during future infectious disease outbreaks and other public health emergencies. Future research might focus on determining which digital-surveillance-related consequences were realized (and with what impact), and which technologies remain in use post-pandemic.

## Data Availability

The raw data supporting the conclusions of this article will be made available by the authors, without undue reservation.
